# Recent Advances in Folates and Autoantibodies against Folate Receptors in Early Pregnancy and Miscarriage

**DOI:** 10.3390/nu15234882

**Published:** 2023-11-22

**Authors:** Xue-Yun Qin, Si-Yao Ha, Lu Chen, Tao Zhang, Ming-Qing Li

**Affiliations:** 1 Laboratory for Reproductive Immunology, Hospital of Obstetrics and Gynecology, Fudan University, Shanghai 200080, China; xyqin23@m.fudan.edu.cn (X.-Y.Q.); 12tsf3004@sina.cn (S.-Y.H.); 2 Assisted Reproductive Technology Unit, Department of Obstetrics and Gynecology, Faculty of Medicine, Chinese University of Hong Kong, Hong Kong 999077, China; 1155187800@link.cuhk.edu.hk; 3 Shanghai Key Laboratory of Female Reproductive Endocrine Related Diseases, Hospital of Obstetrics and Gynecology, Fudan University, Shanghai 200080, China

**Keywords:** folate receptor autoantibody/autoantibody against folate receptor (FRAb), folate receptors (FRs), miscarriage, pregnancy maintenance, trophoblast cells, decidua, macrophages

## Abstract

Though firstly identified in cerebral folate deficiency, autoantibodies against folate receptors (FRAbs) have been implicated in pregnancy complications such as miscarriage; however, the underlying mechanism needs to be further elaborated. FRAbs can be produced via sensitization mediated by folate-binding protein as well as gene mutation, aberrant modulation, or degradation of folate receptors (FRs). FRAbs may interfere with folate internalization and metabolism through blocking or binding with FRs. Interestingly, different types of FRs are expressed on trophoblast cells, decidual epithelium or stroma, and macrophages at the maternal-fetal interface, implying FRAbs may be involved in the critical events necessary for a successful pregnancy. Thus, we propose that FRAbs may disturb pregnancy establishment and maintenance by modulating trophoblastic biofunctions, placental development, decidualization, and decidua homeostasis as well as the functions of *FOLR2*^+^ macrophages. In light of these findings, FRAbs may be a critical factor in pathological pregnancy, and deserve careful consideration in therapies involving folic acid supplementation for pregnancy complications.

## 1. Introduction

Folate, also known as folacin and vitamin B_9_, is involved in folate-mediated one-carbon metabolism (FOCM) and exerts various effects including DNA and RNA synthesis and amino acids metabolism as well as epigenetic modulation [1]. Unlike most bacteria and plants, mammalians are unable to synthesize folate autonomously and, thus, must obtain it from dietary sources. Folate is readily absorbed by the intestine via breakdown from polyglutamates to monoglutamates and subsequently undergoes a series of metabolic processes to be converted into its active form, 5-methyltetrahydrofolate (5-MTHF), before entering the intracellular folate cycle [2]. Functionally, folate metabolism is closely associated with malignancy progression [3], immune function [4,5], and reproductive health [6,7].

Since the 1960s, folate deficiency has been regarded as the major cause of neural tube defects (NTDs). Well-designed studies have proven that folate supplementation could prevent fetal NTDs and ameliorate maternal anemia [8,9]. Consequently, supplementation (0.4 mg/day) is conventionally recommended for women before and during early pregnancy, and folate-fortified food has been mandated by over 50 countries [10,11]. Furthermore, folate is vital for embryo development, implantation, and trophoblast biofunctions. Receiving folate supplements during the periconceptional period could reduce the risk of stillbirth, preterm birth, fetal growth restriction, maternal anemia, and miscarriage [12,13,14,15,16,17,18]; however, too much of a good thing may be bad. Several lines of evidence have demonstrated that a higher dose of folate intake, especially above recommendation, is associated with an increased risk of gestational diabetes mellitus [19] and gestational hypertension [20]. Additionally, nearly 13.6% of miscarriage cases characterized by disturbed folate metabolism could not be rescued with the intervention of single folate supplements [21]. These findings suggest that, due to limited understanding of folate’s effectiveness and regulatory factors during pregnancy, its application in managing pregnancy complications remains challenging.

Folate status can be affected by various factors, including dietary intake, gene polymorphisms, and post-translational modifications of folate transporters and metabolic enzymes. Notably, accumulated evidence has shown that autoantibodies against folate receptors (FRAbs) can significantly disrupt folate internalization by interacting with folate receptors (FRs). FRAbs were first identified in cases of infant cerebral folate deficiency (CFD), in which folate transport from cerebrospinal fluid to the central nervous system is blocked, resulting in relative cerebral folate deficiency, even when peripheral folate status is normal [22]. In addition to CFD, FRAbs have also been implicated in the development of various birth defects and defective behavior, for instance, NTDs and autism spectrum disorders [23,24,25]. Although some studies have indicated an association between FRAbs and subfertility and miscarriage [26,27], there has been little research undertaken to extend our knowledge of the underlying mechanisms. Here, we conducted a review to evaluate folate transport and the formation of FRAbs and to explore their potential roles in pregnancy establishment and maintenance. We then specifically focus on the distribution characteristics of FRs at the maternal-fetal interface and discuss how FRAbs may correlate with miscarriage and impact pregnancy outcomes. Ultimately, we aim to provide insights into therapeutic strategies targeting FRAbs for the management of miscarriage.

## 2. Folate Transport and Metabolism

### 2.1. Folate Transporters

The abundance of intracellular folate is determined by intake and exportation mediated by corresponding transporters, a process which depends on the pH of the internal environment and the specificity of the substrate [28]. Folate is taken up by the proton-coupled folate transporter (PCFT), reduced folate carrier (RFC), and FRs. The PCFT, encoded by SLC46A1, is a high-affinity folate-proton symporter identified in the upper gastrointestinal tract that exclusively transports folates under acidic conditions. The RFC, encoded by SLC19A1, is ubiquitously expressed and serves as the primary tissue folate transporter, bilaterally transmitting a reduced form of folate or its derivative. There are four isoforms of FRs (FRα, FRβ, FRγ, and FRδ) encoded by *FOLR1*, *FOLR2*, *FOLR3*, and *FOLR4*, respectively. FRs are the most important transporters at neutral pH, with high affinity binding to folates in comparison with the RFC and PCFT [29,30]. The α and β isoforms of FRs are membrane proteins anchored by glycosyl-phosphatidylinositol (GPI); they act as metabolism initiators and nuclear transcription factors through the cellular endocytosis of folates. In contrast, the γ isoform lacks a GPI anchor to attach to the cell membrane and works in a secretory manner [28,31,32]. Folate exportation mainly relies on two efflux pumps belonging to the ATP-binding cassette (ABC) superfamily: multidrug resistance protein (MRP) and breast cancer resistance protein (BCRP) [33,34].

### 2.2. Folate Uptake and Mechanism of FOCM

**Figure 1:**
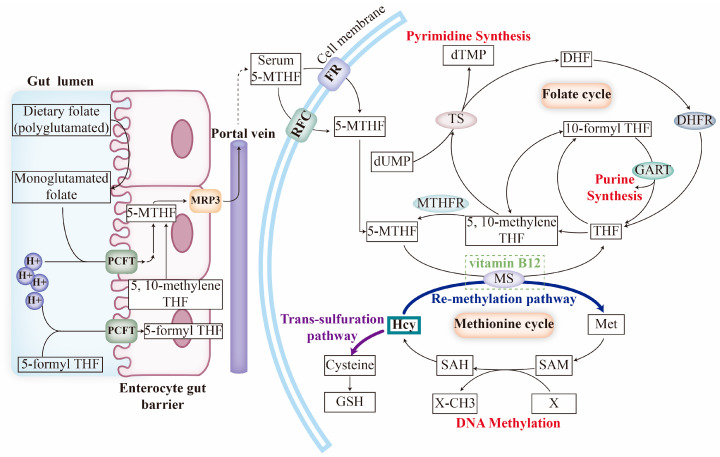
Model graph for folate absorption, cellular uptake, and intracellular folate metabolism. Dietary folate is absorbed by enterocytes via the PCFT and, then, is released into circulation with the form of 5-MTHF. Serum 5-MTHF could be internalized by cells through RFC or FRs and involves an intracellular folate cycle and methionine cycle to exert its effect of pyrimidine, purine synthesis, and DNA methylation. Abbreviations: DHF, dihydrofolate; DHFR, dihydrofolate reductase; dTMP, deoxythymidylate; dUMP, deoxyuridylate; GART, phosphoribosylglycinamide formyltransferase; GSH, glutathione; Met, methionine; MRP3, multidrug resistance protein 3; MS, methionine synthase; MTHFR, methylenetetrahydrofolate reductase; SAH, S-adenosylhomocysteine; SAM, S-adenosylmethionine; THF, tetrahydrofolate; TS, thymidylate synthetase.

Dietary folate is mainly absorbed in the upper small intestine via the PCFT on enterocytes and, then, it is metabolized to 5-MTHF, which is then released into circulation by MRP3. Serum 5-MTHF is transported into cells through their membrane folate transporters, where it participates in FOCM mediated by a series of enzymes [35,36]. During FOCM, intracellular 5-MTHF undergoes the folate cycle and the methionine (Met) cycle (Figure 1).

In the folate cycle, 5-MTHF is initially catalyzed to tetrahydrofolate (THF) via methionine synthase (MS), with vitamin B_12_ acting as a co-factor. THF is then recycled to 5-MTHF in the form of 5,10-methylenetetrahydrofolate (5,10-methylene THF) by methylenetetrahydrofolate reductase (MTHFR). There is also a functional cycle among THF, 5,10-methylene THF and dihydrofolate (DHF), which involves two key events: (1) de novo purine biosynthesis, which is mediated by phosphoribosylglycinamide formyltransferase (GART); (2) de novo dTMP biosynthesis, which is assisted by thymidylate synthetase (TS). In the Met cycle, Met is synthesized from homocysteine (Hcy) via MS then, upon the methyl transfer and modification, Met is converted to Hcy [36,37,38]. It is important to note that Hcy, a pivotal metabolite in the FOCM, can be re-methylated by MS or consumed by the trans-sulfuration pathway. Accumulated Hcy could, to some extent, serve as a biomarker for FOCM inhibition [39,40].

## 3. Classical Viewpoints of FRAbs and Recent Advancements

### 3.1. Hypothesis for Production of FRAbs

**Figure 2:**
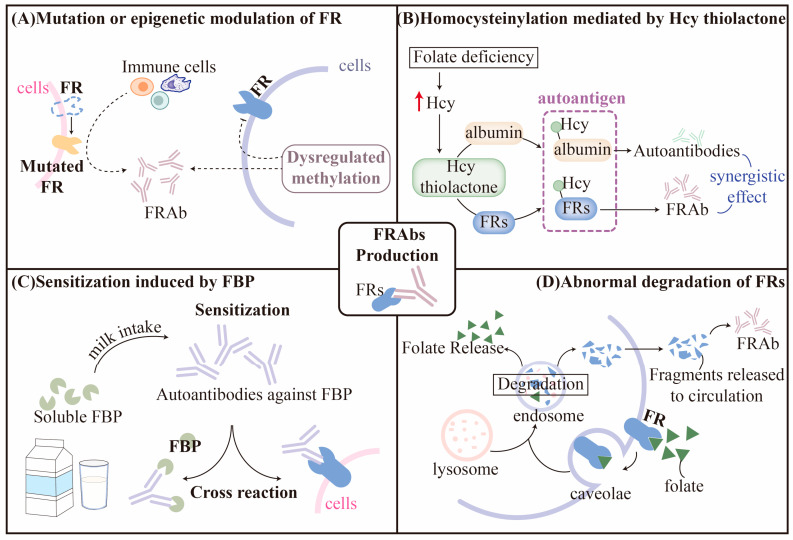
Hypotheses for the production of autoantibodies against folate receptors (FRAbs). (**A**,**B**) On top of gene mutation, aberrant epigenetic modification and homocysteinylation may also alter the antigenicity of FRs to activate autoimmune response. (**C**) Molecular mimicry initiated by soluble folate-binding protein (FBP) could facilitate the production of autoantibodies that cross-react with FRs. (**D**) Abnormal degradation of FRs releases fragments into circulation and newly exposed epitopes may give rise to FRAbs. Dotted arrows represent findings that need further verification. Red arrow stands for elevated level of Hcy.

In general, there have been several classical theories for autoantibodies formation, such as molecular mimicry, newly exposed epitope, aberrant antigen presentation, dysregulated immune network, and so on [41,42]. Interestingly, some of these factors have also been implicated in the generation of FRAbs. Mainstream hypotheses with supported evidence are discussed, respectively, in this section (Figure 2).

#### 3.1.1. Mutation or Epigenetic Modulation of FRs

Receptor mutations are a common cause of autoantibody formation in autoimmune diseases. Mutations and variants of *FOLRs* may alter their antigenicity and have been reported to coexist with blocking FRAb in CFD patients [43]. In one case report, a novel homozygous missense variant of c.524G > T in *FOLR1* was identified in a CFD patient, but the authors did not investigate the FRAb status further [44]; however, according to the whole exome sequencing conducted by Ramaekers et al., no specific mutations of *FOLR1* and *FOLR2* in the coding region were found to be correlated with CFD [45]. Nevertheless, the possibility of multiple unidentified monogenetic causes and X-linked genetic factors cannot be disregarded. Thus, these findings do not fully support the notion that FRAbs result from mutated *FOLRs*.

Epigenetic modification may also contribute to the development of FRAbs. Han et al. reported that missense variants of lysine demethylase 6B (KDM6B) resulted in the decreased expression of KDM6B protein and, thus, dysregulated methylation by upregulating H3K27me2 and downregulating H3K27Ac. This could further inhibit the transcriptional activity of *FOLR1* in an epigenetic way. Interestingly, most participants (5 out of 6 subjects enrolled) who presented missense variants of KDM6B were also FRAb-positive in their serum samples [46]; moreover, considering the role of KDM6B in regulating T-cell differentiation and anti-tumor or autoimmune response [47,48,49], it is possible that KDM6B mutations may be involved in the pathological production of autoantibodies against FRα.

#### 3.1.2. Homocysteinylation of FRs

Epidemiological evidence suggests that the level of FRAbs is positively associated with folate status but negatively associated with Hcy [50]. Depending on the severity of cellular survival stress, low folate status can contribute to hyperhomocysteinemia, which is associated with a range of pregnancy complications such as miscarriage, preeclampsia, fetal growth restriction, and preterm birth [51,52,53]. The mediated underlying mechanism behind the association is thought to be related to abnormal vascular remodeling, acute atherosis, and accelerated maturation of villi, which lead to impaired blood flow and nutrient exchange between the mother and fetus. High maternal Hcy levels around conception have been linked to defective placentation, resulting in smaller placental size and reduced utero-placental vascular volumes during the first trimester of pregnancy. Interestingly, this negative association was stronger in women who conceived after undergoing the treatment of in vitro fertilization/intracytoplasmic sperm injection (IVF/ICSI). This could be because hormone stimulation during IVF/ICSI treatment can lead to increased oxidative stress and inflammation, which can further exacerbate the negative effects of high Hcy levels on the placenta [54]. Some studies, however, have shown no association between miscarriage and elevated Hcy [55,56], possibly due to different population baselines, dietary habits, or adaptive metabolism mediated by the high level of Hcy. Hcy can be metabolized through the re-methylation or trans-sulfuration pathways, or it can be transformed into a reactive thioester catalyzed by the enzyme methionyl tRNA synthetase to avoid misincorporation of Hcy [57,58,59]. If folate or vitamin B_12_ deficiency inhibits the remethylation or trans-sulfuration pathways, Hcy is more likely to form an active Hcy thiolactone (HTL), which can homocysteinylate proteins by interacting with lysine residues and cause structural abnormalities and loss of protein function [59]. Animal studies have shown that HTL-modified proteins are immunogenic and can increase the titers of specific autoantibodies that recognize the epitope of Hcy-εN-lysine in proteins. Autoantibodies against homocysteinylated hemoglobin or albumin are common in humans and are positively correlated with Hcy status [60]; moreover, HTL can induce ER stress and inflammation response [61].

It is interesting to note that FRs contain high levels of lysine residues, which suggests the possibility that they could be modulated by HTL and potentially misidentified as autoantigens [62]. This could lead to the activation of autoimmunity and the production of FRAb [62,63]. This theory is supported by an in vivo study conducted by Denny et al., where pregnant mice, fed on folate-deficient diets, showed a negative correlation between maternal folate status and Hcy levels, and the presence of autoantibodies to homocysteinylated proteins was detected [64]; however, further studies are required to confirm this hypothesis and to determine the mechanisms linking homocysteinylation with autoimmunity.

Homocysteinylation mediated by HTL has also been observed in other proteins related to FRAb function. For example, Tang et al. demonstrated that excessive Hcy induced by folate deficiency may lead to increased homocysteinylation of heterogeneous nuclear ribonucleoprotein E1(hnRNP-E1), which interacts with its mRNA cis-element and forms a positive feedback loop, eventually resulting in accumulation of hnRNP-E1. hnRNP-E1 has a high affinity to an 18-nucleotide cis-element in the 5′-untranslated region of FRα mRNA, which triggers proportionate translational upregulation of FRs [65]. This research expands our knowledge of the compensatory upregulation of FRs in the context of FRAbs and folate deficiency. Additionally, abnormal inflammatory response has been found to induce anti-homocysteinylated albumin autoantibodies that can play a synergistic role with FRAbs, leading to diseases complicated by an increased risk of vascular events or hypercoagulability such as miscarriage [66,67].

#### 3.1.3. Sensitization Induced by Folate-Binding Protein (FBP)

As a soluble form of FR, FBP is primarily present in human or bovine milk and shares approximately 80–90% homology with the amino acid sequence of membrane-anchored FRα and FRβ in the human placenta [68,69]. The FRs cross-react with antibodies against human milk folate-binding protein, and the antiserum to human milk FBP localizes in the syncytiotrophoblastic layer of the villi in the first trimester [70]. Sensitized by FBP with similar epitopes from dairy products, FRAbs may initially be generated within the intestinal wall and, then, distributed throughout the body via circulation. Interestingly, a milk-free diet could downregulate autoimmunity and significantly decrease FRAbs titers [71]; however, after the re-introduction of bovine milk for 3–6 months, the titers of FRAbs rose again [72]. Notably, this hypothesis could also partially explain the fluctuating levels of FRAbs observed clinically [73]. As proposed by Ramaekers et al., newly generated FRAbs may firstly block the binding sites of antigens from FBP, thus reducing the interaction between FRs antigens and B lymphocytes, which leads to a reduction in FRAb levels; however, with the removal of FR-FRAbs complex in the intestinal lumen, FRAb production could recuse, causing the levels to fluctuate in a cycle-dependent manner [45].

#### 3.1.4. Abnormal Degradation of FRs

Certain membrane-anchored receptors such as FRs, the follicle-stimulating hormone receptor, and the thyrotropin receptor, exert their effects via internalization through a process named receptor-mediated endocytosis [74,75]. Upon binding to ligands, receptors undergo conformational alterations and interact with endocytic proteins, resulting in rapid internalization and the formation of caveolae on the cell surface. Vesicles are then sorted into different compartments for recycling or degradation via the endosomal pathway [75]. Although the underlying mechanism remains elusive, it has been proposed that the abnormal degradation of receptors may expose new epitopes or antigenic determinants recognized by the immune system as foreigners, leading to an autoimmune response [76,77,78]. The degradation of membrane-anchored receptors may also release peptides into circulation, potentially stimulating an immune response in the secondary lymphatic tissues. Studies have shown that the aberrant degradation of follicle-stimulating hormone receptors and thyrotropin receptors may elicit relevant autoantibodies in this way, interfering with the specific signaling pathway in which they are involved [78,79]. Likewise, Knutson et al. predicted potential epitopes derived from FRα degradation, which could activate and stimulate CD4^+^ T cells, accompanied by increased levels of certain antibodies against FRα [80]. These results imply that, if internalized FRs undergo abnormal degradation, the neo-epitopes of specific peptides may be exposed, potentially leading to their recognition by T cells and the subsequent production of FRAbs.

It is important, however, to note that other factors, such as genetics or immune activation, may also induce the production of FRAbs. For example, studies have reported an association between genetic variations in FOCM-related enzymes, including *MTHFR* (rs1801133, C → T), *DNMT3A* (rs7560488 C → T), and *MTHFD2* (rs828903, A → G), and elevated plasma levels in FRAbs [81]; nevertheless, the causal relationship between these factors and FRAbs remains undetermined. Recent studies have attempted to link FRAb-positive autism spectrum disorders, and miscarriage or infertility, with immune-related genetic defects of MHC genes, such as variants of HLA-B/DQB1/DQ2 [82,83]. Further research is required to elucidate the association between MHC polymorphisms and FRAb status, as well as the relevant mechanisms.

### 3.2. Classification and Characteristics of FRAbs

FRAbs can be classified into blocking and binding subtypes based on their function. Blocking FRAbs refer to autoantibodies that share the same binding sites as folates and inhibit the combination of folates with FRs. Conversely, binding FRAbs recognize different binding sites from folates and, therefore, do not compete with folates but, instead, inactivate FRs by triggering an immune response and inflammation through antibody-dependent cellular cytotoxicity (ADCC) or antibody-dependent cellular phagocytosis (ADCP) [72,84,85]. Thus, the functional binding assay of ^3^H-folic acid and the ELISA method can be used to distinguish blocking and binding FRAbs, respectively, according to their properties [72]. The functional blocking of folate transport and inflammation induced by FRAbs are interrelated in the disease pathology [35]. Frye et al. found the severity of autism varied among patients whose predominant FRAbs were binding or blocking subtypes. These two groups responded differently to folic acid treatment. [86]. Besides, blocking rather than binding FRAbs were negatively correlated with thyroid function (evaluated by Thyroxine/TSH) in a dose-response manner [87]. These findings suggest that different phenotypes of FRAbs affect disease development and therapeutic effects to varying extents.

Minor differences in the heavy chain of immunoglobulin distinguish the IgG and IgM isoforms of FRAbs in different situations. For instance, IgG1 and IgG2 predominated in FRAbs found in pregnant women with NTDs, while IgG1 and IgG4 isotypes constituted the majority of serum FRAbs in CFD children, with an average biological half-life of 21 days [45,88]. Cabrera et al. collected maternal serum from pregnant women with NTDs and normal controls during gestational weeks 15–18 for FRAb separation. They found FRAbs against bovine FBP were mainly IgM, whereas those against human placental FRs were a mixture of IgG and IgM. Interestingly, they also observed that FRAb status did not consistently correlate with the extent of inhibited folate binding in all cases [85], implying that mixed FRAbs may recognize different epitopes from FBP and FRs respectively. In other words, only FRAbs induced by a given epitope could compete with folate for binding.

## 4. Association between Folate or FRAbs and Miscarriage

### 4.1. Folate Deficiency and Miscarriage

In addition to limited folate intake and elevated endogenous Hcy [89,90,91,92,93], genetic mutations in metabolic enzymes or folate transporters can also lead to folate deficiency due to dysregulated FOCM or impaired folate utilization, which have already been identified in miscarriage cases. For instance, as the most common variations of MTHFR, missense mutations of C677T and A1298C reduce its metabolic activity and are associated with an elevated risk of miscarriage [94,95,96]. Besides, in terms of folate transporters, heterozygous deletion or missense mutation of *FOLR1* (encoding FRα) and *SLC19A1* (encoding RFC) have been found to be associated with an increased risk of recurrent miscarriage [97,98,99].

However, the association between miscarriage and the mutation of enzymes or folate transporters at different loci are inconsistently demonstrated in previous studies [56,97,100,101,102,103]. These inconsistencies might be attributed to several variables, including differences in sample size, ethnicity, genotyping methods, heterogeneity of miscarriage pathology, and the presence of confounding factors, such as maternal age, lifestyle, hormonal imbalances, and environmental exposures. Despite attempts to reconcile these inconsistent results, there is no convincing and widely accepted explanation yet. To shed light on this issue, further investigation is needed to elucidate the upstream regulatory factors involved in folate internalization and FOCM such as FRAbs.

### 4.2. Expressional Profile of FRs at Maternal-Fetal Interface

The maternal-fetal interface is primarily composed of the maternal decidua and the maternal side of the placenta; it plays a critical role in promoting proper fetal growth and development while maintaining a healthy pregnancy. As the target of FRAbs, FRs present a specific expressional profile at the maternal-fetal interface (Table 1).

FRα, encoded by *FOLR1*, is a high-affinity FR anchored to the cell membrane via GPI. FRα exhibits a restricted distribution pattern in normal tissues [118], with expression primarily limited to a polarized epithelia in various organs such as the choroid plexus, kidney, fallopian tube, uterus, ovary, lung, and placenta [26,119,120,121]. At the human maternal-fetal interface, the positive staining of FRα is localized to the apical surface of the syncytiotrophoblast and decidual glandular epithelial cells; there is also occasional positive staining within the stroma of human decidua [29,84,104,105,106,107,108]. This localization facilitates the transfer of folic acid to the fetus before maternal-fetal circulations are fully established at the end of the first trimester. FRα is present throughout the entire gestation period, but its expression gradually decreases as the first trimester progresses into the second [122,123]. Functionally, FRα is the predominant transporter responsible for placental folate uptake via receptor-mediated endocytosis, accounting for approximately 60% of folate transport in the placenta [28,105]. In mice placentae, FRα is expressed in the labyrinth layer, with positive staining observed in syncytiotrophoblasts, trophoblast giant cells (E8.5 onwards), glycogen trophoblast cells (E12.5–E18.5), and their progenitors. The expression of FRα peaks between E9.5 and E12.5 when the labyrinth undergoes significant morphogenesis but, then, it declines as gestation progresses [109], indicating that a folate uptake mediated by receptors may be required for placental development. Likewise, staining of FRAbs against FRα was mainly observed in the syncytiotrophoblasts and cytotrophoblasts of rat placenta and decidual cells [26,110], and FRα expression was gradually increased from 14–20 days of gestation [105].

FRβ, which shares 70% identity with FRα in homology, is encoded by *FOLR2*. It has been reported to primarily express on activated macrophages and dendritic cells, while resting macrophages are devoid of FRβ expression under physiological conditions [5,124,125,126,127]. At the maternal-fetal interface, FRβ mainly exists in decidua [108] and localizes on the surface of macrophages derived from human placentae [7,84].

FRγ, encoded by *FOLR3*, is a soluble protein that lacks a GPI anchor domain and originates from the secretory granules of neutrophil granulocytes. It has a much lower affinity for folate than FRα and FRβ [111]. Some studies have suggested the potential roles of FRγ in regulating stem cell senescence [112], inflammation [113], and anti-tumor response induced by chemotherapy [114]. The role of FRγ in pregnancy remains unclear because neutrophils are rare at the maternal-fetal interface under normal physiological conditions; however, FRγ may be involved in implantation, placentation, tissue remodeling, and mediating immune tolerance by interacting with T cells or innate lymphoid cells [128,129]. In pathological conditions like septic miscarriage, there is notable infiltration of neutrophils in decidua [130]. More research is needed to determine whether and how FRγ might regulate inflammatory responses or immune tolerance mediated by neutrophils.

FRδ, also named Izumo1 receptor (Izumo1R) and encoded by *FOLR4*, is a pseudo folate receptor that cannot transport folates. It was initially identified in sperm cells and is responsible for fertilization via interaction with its ligand, Izumo1, on oocytes of several mammalian species, including humans and mice [131]. Besides, recent studies have suggested the expression of FRδ and its ligand at the surface of Treg cells and γδT cells, respectively [115,116,117]; however, whether FRδ and its ligand express on decidual T cells at the maternal-fetal interface remains elusive.

### 4.3. FRAbs and Miscarriage

FRAbs target the placenta and embryos, and have been shown to inhibit folate transport from rats to their fetuses, leading to folate deficiency during pregnancy [110,132]. Da Costa et al. demonstrated that the intraperitoneal administration of a low dose of antiserum to FRs, on the 8th day of gestation, increased the embryo resorption rate in rats, which could be rescued by folinic acid; however, it was dexamethasone instead of folinic acid that reversed the embryo-lethal effect induced by a larger dose of antiserum [26]. This finding suggests the major pathological factor caused by a larger dose of antiserum is a hypersensitivity reaction rather than a single folate deficiency. Although the antiserum used was polyclonal and unpurified, obtained from human placental FRs that were a mixture of antibodies against both FRα and FRβ, these results might provide preliminarily insight into the relationship between FRAbs and miscarriage. Further investigation is required to elucidate the mechanism by which FRAbs targeting different FR isoforms are involved in pregnancy.

Initially, epidemiological evidence revealed a link between miscarriage and FRAb-related birth defects, like NTDs, in human pregnancy [133,134]. Women with a history of miscarriage, particularly those complicated with elevated FRAbs, were found to have a higher risk of NTDs in subsequent pregnancies [133]; moreover, positive results from serum-screening tests for NTDs were associated with adverse pregnancy outcomes, including miscarriage [134]. In one case report, a pregnant woman with a history of infertility and multiple miscarriages was positive for FRAbs. She conceived and had a successful pregnancy with the usage of d, l-leucovorin, a milk-free diet, and a low dose of prednisone during pregnancy [135], which highlights the potential role of FRAbs in miscarriage as well as the feasibility of therapeutic methods targeting FRAbs. Large-scale multicenter studies are still, however, required to confirm this association, and it is important to note that the blocking or binding subtypes of FRAbs might have different effects on pathogenesis. For example, according to Berrocal-Zaragoza et al., blocking FRAbs were found to be positive in 29.4% of subfertility cases with varying degrees of titer fluctuation; this predisposed individuals to a twelve-fold higher risk of subfertility compared to negative participants [27].

### 4.4. Implications of FRAb Production-Related Risk Factors in Miscarriage

Based on the hypotheses we discussed above, several risk factors have been implicated in facilitating FRAb production in miscarriage. These include aberrant expression, the modulation or degradation of FRs, homocysteinylation mediated by Hcy thiolactone (HTL), and FBP-induced sensitization. In complicated cases of miscarriage, limiting FBP intake through a milk-free diet has shown to have an adjuvant therapeutic effect on reducing FRAb titers [135,136]. Interestingly, both Hcy and its active form for homocysteinylation, HTL, have been reported as risk factors for both miscarriage and FRAb production, particularly in reproductive failure characterized by hypercoagulable status [66,67]. Additionally, studies have shown a link between genetic variations of *DNMT3A* (rs7560488 C → T) and the elevated plasma levels of FRAbs [81]. In fact, defective decidualization, with reduced implantation sites and the loss of embryos during pregnancy, has been observed in mouse models of folate deficiency. This may be due to the downregulation of DNA methylation via inhibition of *DNMT1* and *DNMT3* [91,92,93], indicating a potential association between abnormal methylation and FRAbs in miscarriage. This warrants further investigation.

Other factors that could lead to the unexpected exposure of FR epitopes at the maternal-fetal interface may also contribute to the production of FRAbs. Rothenberg et al. proposed that clinically unrecognized pregnancies, known as biochemical pregnancies, could partially explain the high incidence of spontaneous miscarriage concurrent with the presence of FRAbs in apparently healthy individuals [137]. The risk is probably due to slight injury and proteolysis of the reproductive tissues, which can lead to residual trophoblast cells being exposed to the maternal immune system, resulting in FRAb production [44]. Further research is needed to investigate the mechanism underlying FRAb production and its correlation with pregnancy complications such as miscarriage, particularly with regard to any potential immunity or genetic risk factors. Existed evidence that support the association between FRAbs and miscarriage is summarized in Table 2.

## 5. Regulatory Effects of FRAbs against FRα (FRαAb) on Trophoblast Cells and Decidua

### 5.1. FRαAb and Trophoblastic Biofunctions

Trophoblast cells, which originate from the outer layer of blastocysts, play a vital role in placentation. They differentiate into villous trophoblasts (VTs) and extravillous trophoblasts (EVTs); however, impaired trophoblastic biofunctions and placentation can lead to pregnancy complications such as miscarriage. Since FRα is responsible for over 60% of folate transport in trophoblast cells, and plays a crucial role in determining intracellular folate status [28,105], it is rational to speculate that FRAbs may interfere with the bio-behaviors of trophoblast cells mediated by FRα. Although related studies are limited, we aim to depict the role of FRAbs based on a discussion of how folate status regulates trophoblastic functions by interacting with FRα.

#### 5.1.1. Viability and Proliferation of Trophoblast Cells

Several studies have confirmed the role of FRAbs in inhibiting cellular folate uptake and interfering with FOCM, particularly in cases of CFD or NTDs [43]. These conditions are often associated with elevated levels of Hcy, which is also seen in many pregnancy complications characterized by relative folate deficiency [88,138]. Induced folate deficiency and Hcy have been shown to elicit apoptosis in human trophoblast cells derived from placentae and to reduce their hormone production, probably by triggering DNA damage. These effects could be rescued by antioxidants or folates [139,140]. Additionally, research on EVTs from human placenta explants has indicated that apoptosis is activated in 10^−6^ M culture condition of folate, but inhibited in 10^−8^ M and 10^−10^ M [141], suggesting that the role of folate status in trophoblastic viability might work in a concentration-dependent manner; moreover, for human placental cell lines in vitro, either folate shortage or an excessive dose (2000 ng/mL) has been shown to impair viability and induce apoptosis of JEG-3, HTR-8 and BeWo [142,143], which exhibit differentiated characteristics. For instance, BeWo has demonstrated an overt edge over HTR-8 for self-proliferation and capacity for the enrichment of folates at the same concentration. A high dose of folic acid (2000 ng/mL) reduced trophoblastic viability in BeWo but promoted cellular proliferation in the HTR-8 [143], which might be attributed to the different relative expressional profiles of FRs in other folate transporters [123]. It would be interesting to see further studies applying FRAbs or designed monoclonal antibodies against FRα, exploring their effects on trophoblastic viability.

#### 5.1.2. Placental Development

Folic acid plays a key role in the early stages of placental development, including EVT invasion, angiogenesis, and secretion of the matrix metalloproteinases (MMPs) [144]. Periconceptional folic acid supplement intake has been linked to lower resistance of blood flow and improved nutrition support of the placenta in the second and third trimesters [145]; therefore, folate is involved in placental development during early pregnancy, and regulates and maintains placental function throughout pregnancy.

FRAbs might disturb placentation in a metabolic-dependent way. Firstly, by inhibiting intracellular folate bioavailability and reducing MMP production or by inducing oxidative stress, folate deficiency caused by FRAbs has been shown to dysregulate trophoblastic migration and invasion [141,142,143,146,147]. Secondly, FRAbs may restrict the activation of FOCM and downstream epigenetic modification by blocking FRα. In placental tissue, folate supplementation can promote the methylation of growth-promoting genes and is subsequently downregulated as gestation progresses [148]. The peroxisome proliferator-activated receptor (PPAR) is a ligand-dependent transcriptional regulator that works with the peroxisome-proliferator response element (PPRE) to regulate the transcriptional activity of target genes [149]. Maternal malnutrition, such as folate deficiency, may elevate Hcy levels and decrease PPAR expression in the placenta, leading to reduced placental angiogenesis, trophoblasts differentiation and invasion, and adverse pregnancy outcomes such as miscarriage and preeclampsia [150]. It is unclear, however, whether this effect is directly mediated by increasing pro-angiogenesis factors or indirectly by regulating EVT invasion [141,142,143,146]. Although Meher et al. reported no significant morphological alterations of placentae in rats fed with a folate-deficient diet, increased oxidative stress, reduced PPAR levels, and elevated TNF-α and IL-6 were all observed. These effects could be partially reversed by folate supplementation [151]. Further research is needed to investigate whether folate modulates PPAR expression in an epigenetic manner involving FRα and FRαAb.

### 5.2. FRαAb and Decidual Homeostasis

The process of decidualization involves the transformation of secretory-phase endometrium into decidua, which is stimulated by estradiol (E2) and progesterone (P4). It prepares the uterus for successful implantation, embryonic development, and pregnancy maintenance [152,153]. During this process, elongated and fibroblast-like endometrial stromal cells (ESCs) differentiate into rounded and epithelioid-like decidual stromal cells (DSCs) [154], resulting in the production of prolactin (PRL) and IGFBP-1. In addition, endometrial epithelial cells (EECs), the outermost layer of cells within the endometrium, are highly responsive to hormones and play a vital role in regulating decidual homeostasis [155] by interacting with DSC and decidual immunocytes [156,157]. FRα is mainly located in EECs and occasionally in DSCs in human decidua [104]. The same findings have been observed in rats [26,110], indicating that FRAbs may inhibit decidualization and disrupt decidual homeostasis.

#### 5.2.1. Decidualization

It is believed that FRαAb blocks or inhibits the uptake of folate by EEC, as FRs mainly express on EECs in decidua. A deficiency of folate in mice has been shown to result in the defective progression of DSC decidualization, probably through the modulation of genomic methylation or a decrease in E2 and P4 hormones [91,92]; however, there have been no follow-up studies exploring the relationship between FRAbs and EEC in decidualization. FRα is a ubiquitously upregulated marker in a wide range of epithelial tumor cells and may play a role in promoting cell growth and malignancy progression through various mechanisms [158,159,160]. Blocking FRα may impair DNA synthesis, energy production, and cell proliferation by inducing deficient folate intake and inhibiting FOCM. In early pregnancy, FRαAb is speculated to work in this way because folate is metabolically activated to meet the demands of EECs or DSCs for dramatic matrix remodeling and cell proliferation during decidualization [91,92]. Additionally, FRα has been found to serve as a signaling molecule to regulate its downstream JAK-STAT3 pathway by binding with a membrane co-receptor, i.e., gp130 [161]. Our previous research has shown that decidualization can be manipulated by a gp130/IL27RA-STAT3-ESR/PGR axis [162], indicating that FRαAb may interfere with FRα-mediated STAT3 signaling and, thus, inhibit decidualization.

Folate has also been shown to attenuate LPS-induced increases in COX-2 in mouse placentae [163]. As COX-2 plays a positive role in decidualization [164,165], FRα signaling may be a regulator that balances COX-2 for optimal status during pregnancy. Notably, certain crucial genes associated with the cellular cycle (*Trp53*), differentiation (*Nr1h3*, *Nr5a1*), and angiogenesis (*Ereg*), were hypermethylated and therefore downregulated in the decidua [92]. Furthermore, folate deficiency led to elevated Hcy levels and impaired decidual angiogenesis by downregulating pro-angiogenic factors including *VEGFA*, *VEGFR2*, and *PLGF*, resulting in a poorly vascularized decidua [91]. These studies indicate that FRAbs may regulate decidual angiogenesis by interfering with methylation. Further research is needed to investigate regulated genes and their functional effects induced by FR mutations or FRAb treatment through transcriptome sequencing.

#### 5.2.2. Decidual Apoptosis and Autophagy

In contrast to E2 and P4 or a combination of the two, the cAMP-induced decidualization of DSCs in vitro can produce soluble pro-apoptotic factors that might be critical for decidual remodeling and involution during pregnancy [166]; however, folate deficiency has been shown to reduce decidual apoptosis and the level of ROS in the endometrium by inhibiting the mitochondrial apoptosis pathway, which also leads to impaired decidualization with reduced protein markers, including BMP2, Hoxa10, and MMP2 [167,168].

Autophagy, a highly conserved cellular process responding to survival stress, has recently been found to regulate menstruation, placentation, decidualization, and maternal immune tolerance during pregnancy. Both in vivo and in vitro studies have demonstrated that autophagy is suppressed under folate-deficient conditions by aberrantly activating AMPK/mTOR, which results in impaired decidualization [167,169]. Interestingly, this process could be rescued by rapamycin, an inducer for autophagy [169]; therefore, in addition to folate supplementation, therapeutic approaches targeting autophagy could complement the treatment of any defective decidualization induced by folate deficiency or impaired folate bioavailability resulting from FRαAb.

## 6. Regulatory Effects of FRAbs against FRβ (FRβAb) on Regulating Decidual Macrophages (dMφ)

Recent studies have found that FRβ is expressed in inflammatory or autoimmune diseases and tumor-associated macrophages (TAMs), and it may be regulated by its positive transcriptional factor PU.1 [170]. FRβ expression may be involved in the origin, polarization, and immune regulation of macrophages; however, the presence of FRβAb can interfere with these effects by blocking folate uptake and causing antibody-dependent cellular cytotoxicity (ADCC) or antibody-dependent cellular phagocytosis (ADCP) [84]. Feng et al. have developed a monoclonal, artificially designed antibody specific for human FRβ, which was able to mark the activated macrophage and mediated cytotoxicity by ADCC [124]. This shows potential in eliminating aberrantly activated macrophages and monocytes in autoimmune diseases; nevertheless, there has been no research on the effects of FRβAb on the function regulation of dMφ during pregnancy. Studies have shown that *FOLR2* expression accounts for the maturation and enrichment of macrophages, and that *FOLR2*^+^ macrophages may be involved in regulating cancer progression, immune response, and angiogenesis (Table 3). This provides insight into the potential role of FRβAb in maintaining pregnancies involving dMφ.

### 6.1. FRβAb and Origin of dMφ

According to classical viewpoints, dMφ are derived from peripheral monocytes that originate in bone marrow; however, recent hypotheses propose that the embryonic precursors of macrophages, pluripotent CD34^+^ stem cells in situ, and tissue-resident macrophages prior to pregnancy, could also be potential origins [193]. Interestingly, the expression of FRβ in hematopoietic stem cells (HSCs) has been identified, although it is unable to elicit any detectable folate transport in HSCs. FRβ is upregulated in CD34^+^ cells during cell differentiation, particularly among subgroups of monocyte–macrophage precursors such as CD34^+^CD15^+^ and CD34^+^CD38^+^ cells [171]. Polyclonal FRAbs have consistently been shown to increase the cloning efficiency of colony-forming unit-granulocyte macrophages, which is a cell precursor that resides in bone marrow [172]. These findings indicate that FRAbs targeting FRβ might be involved in the origin of dMφ by balancing self-renewal, proliferation, and differentiation, although this requires further verification. In fact, *FOLR2*^+^ macrophages have been reported to secrete a series of inflammatory cytokines and chemokines, such as IL-8, CCL2, CCL3, and CCL4 [127]. Among these cytokines, CCL2, CCL3, and CCL4 are essential regulators for recruiting TAMs from peripheral monocytes [173,174,175], indicating a positive regulatory loop between tissue-resident *FOLR2*^+^ macrophages and newly recruited ones. It would be fascinating to investigate whether and how FRβ and its autoantibodies regulate dMφ populations by regulating cell recruitment.

### 6.2. FRβAb and dMφ Polarization

Macrophages can be categorized into two main phenotypes based on their functional characteristics: M1 (CD80^+^CD86^+^CD11c^+^) and M2 (CD163^+^CD206^+^) macrophages. Resting macrophages are polarized to M1 phenotype macrophages when stimulated by lipopolysaccharide (LPS), IFN-γ, or a granulocyte macrophage colony-stimulating factor (GM-CSF). M1 macrophages, on the one hand, produce pro-inflammatory cytokines like IL-12 and TNF-α. M2 macrophages, on the other hand, can be induced by IL-4, IL-10, or M-CSF, and produce anti-inflammatory factors IL-10 and TGF-β [194,195]. M1-like macrophages are involved in tumor suppression and resistance to pathogens; M2-like macrophages actively participate in tissue repair, remodeling, angiogenesis, and immune regulation [196]. Although there is generally a mixture of M1 and M2 phenotypes in decidua, M2 has been widely acknowledged as the predominant phenotype of dMφ during early pregnancy, after implantation [197]; however, to date, no research has demonstrated the expression pattern of FRβ during dMφ polarization throughout gestation.

FRβ is preferentially expressed on human anti-inflammatory M2, induced by M-CSF macrophages, and in M2-polarized TAMs. The expression of FRβ correlates with an increased ability to uptake folate. CD163^+^ TAMs are FRβ-positive and dependent on M-CSF [176]. FOLR2 was found to be expressed in TAMs with specific involvement in its M2 polarization, and it is a prognostic factor for bladder cancer [198]. Interestingly, FRβ exhibits a particular expressional profile based on different stimulative conditions or regulators. For instance, FRβ remains high on M2 macrophages and triggers significantly increased folate uptake in the context of LPS or IFN-γ stimulation [177,178]. The repolarization of M2 towards M1 macrophages, mediated by GM-CSF, leads to the upregulation of CD206 and *SLC19A1*, and the downregulation of *FOLR2* via activin A-activated SMAD signaling [7,179,180]. Conversely, anti-inflammatory treatment by corticosteroids tilts GM-CSF macrophage differentiation toward an M2 and FRβ-positive phenotype [178]. Conditional knockout mice with CD40 deletion in macrophages presented a higher content of a subgroup of CD206^+^ tissue-resident M2 macrophages, with significant upregulation of *Folr2* detected by RNA sequencing [199]. Clearing FRβ^+^ macrophages with anti-FRβ immunotoxin could inhibit the proliferation of activated macrophages and fibrosis mediated by M2-like macrophages [126,181], due to the potential of FRβ in exhausting various fibrotic factors secreted by inflammatory macrophages. These findings imply that FRβAb may be involved in miscarriage and preeclampsia by disturbing the phenotype of dMφ polarization during pregnancy. This is because aberrantly polarized dMφ have been shown to be associated with the development of certain pregnancy complications characterized by the dysregulation of decidual homeostasis [200,201,202].

### 6.3. FRβAb and dMφ-Mediated Angiogenesis

*FOLR2*^+^ TAMs reside in a perivascular niche within the tumor stroma [182] and secrete pro-angiogenic cytokines such as TGF-β and VEGF [183]. Thomas et al. identified a unique population of fetal macrophages expressing *FOLR2*, i.e., Hofbauer cells (HBCs), in the human placental stroma during the first trimester. HBCs secrete VEGFA, osteopontin (OPN), the tissue inhibitor of metalloproteinase 1 (TIMP-1), and MMP-9 to promote angiogenesis and vascular remodeling in a paracrine manner [127]. Notably, OPN is of great significance in placental development, decidualization, and pregnancy maintenance due to its diverse roles in enhancing EVT invasion, the phenotype switching of vascular smooth muscle cells, and the regulation of decidual immune cells. In synergy with MMP-9, OPN may also mediate crosstalk between dMφ and trophoblast cells [184]. These results illustrate the role of *FOLR2*^+^ macrophages in regulating decidual angiogenesis and vascular remodeling; however, the regulatory mechanisms of *FOLR2* that facilitate angiogenesis and vascular remodeling are still unclear. Further studies that intervene with *FOLR2* expression and investigate correlated signaling pathways could assist in understanding the mechanisms involved.

### 6.4. FRβAb and dMφ-Mediated Immune Regulation

*FOLR2* has long been recognized as a marker for the immunosuppressive phenotype of tissue-resident macrophages. In human hepatocellular carcinoma, the overexpression of CD68 and CD163 in *FOLR2*^+^ TAMs have been associated with a worse prognosis [203]. To reveal the underlying mechanisms, Sharma et al. demonstrated that *FOLR2*^+^ TAMs mediate immunotolerance in hepatocellular carcinoma by interacting with Foxp3^+^ Treg cells through CD86/CTLA4 interaction and crosstalk with endothelial cells mediated by NOTCH signaling [185]. Additionally, *FOLR2*^+^ TAMs can maintain immune tolerance in situ by secreting immunosuppressive cytokines such as IL-10 [176,186]. In vitro studies have shown that *FOLR2*^+^ macrophages co-express CD39 and CD73, which boosts immunosuppression by activating the purinergic pathway, releasing extracellular ATP and converting it to immunosuppressive adenosine, thereby inhibiting the activity of CD4^+^ T cells [187]. The balance of adenosine and inosine is regulated by the folate pathway, which modifies the cytotoxicity, proliferation, and immune status of NK cells [188]. These findings suggest that *FOLR2* and its downstream adenosine play a role in mediating mutual immune regulation among macrophages, NK cells, and CD4^+^ T cells.

On the other hand, artificially inhibiting *FOLR2* may impair immune tolerance. Treating mouse peritoneal macrophages with CpG-DNA, combined with an anti-IL-10 receptor (IL-10R) antibody, can upregulate MHCII, IL-2, IL-6, and IL-13 molecular expression while suppressing *FOLR2*, MMP-9, and VEGF, and can reverse M2 polarization to M1. Additionally, research has demonstrated a significant reduction in detectable lung-metastasis foci in vivo, suggesting that targeting IL-10R could alter the immune tolerance established by *FOLR2* [189]. Specifically targeting FRβ with chimeric antigen receptor (CAR)-T resulted in the elimination of FRβ^+^ TAMs, an enrichment of pro-inflammatory monocytes, an influx of cytotoxic CD8^+^ T cells, the inhibition of tumor progression, and better prognosis in a mouse model [190]. Therapeutically targeting *FOLR2* M2-like macrophages can increase the proportions of M1 macrophages and CD3^+^ T cells; however, it has no effect on CD4^+^ and CD8^+^ T cells [191,192]. Overall, these studies suggest that *FOLR2*^+^ macrophages play a major role in mediating immune tolerance.

Some studies, however, have challenged this point of view and added complexity to the roles of the *FOLR2*^+^ macrophage in immune regulation. For instance, although fetal macrophages in placental stroma are confirmed to be *FOLR2*-positive and highly express CD206, they also demonstrate an active response to Toll-like receptor stimulation and possess active microbicidal capacity [127]. Nalio et al. identified a distinct population of *FOLR2*^+^ macrophages in the immune microenvironment of breast cancer that was colocalized with CD8+ cytotoxic T lymphocytes (CTLs) adjacent to lymphoid tissue. Surprisingly, they found that *FOLR2^+^* TAMs in breast cancer were MHCII-positive and expressed the gene signatures of both M1 and M2 simultaneously. These macrophages were capable of activating CTLs via long-lasting interaction, contributing to the antitumor immune response [182]. Further studies are needed to explore the underlying mechanisms by which *FOLR2* expression on macrophages regulates the intricate immune landscapes of the microenvironment through crosstalk with other lymphocytes in situ.

## 7. Summary and Future Perspectives

**Figure 3:**
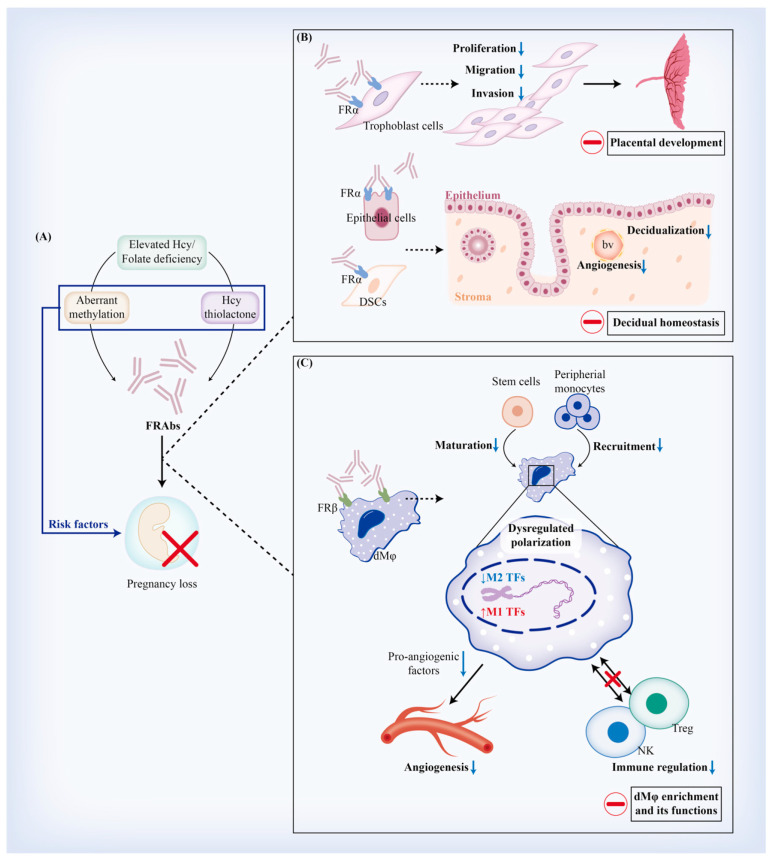
Potential mechanisms by which FRAbs lead to miscarriage. (**A**) Relative folate deficiency and increased Hcy status may be involved in FRAb production through Hcy thiolactone and aberrant methylation modification, which have been recognized as risk factors for miscarriage. (**B**) FRAbs against FRα may interrupt placentation and decidual homeostasis via binding with FRα on trophoblast cells, epithelial cells, or decidual stromal cells. (**C**) FRAbs could also interact with FRβ on decidual macrophages and dysregulate its maturation, recruitment, and polarization. Besides, angiogenesis and immune regulation mediated by macrophages might also be impaired. Abbreviations: bv, blood vessels; dMφ, decidual macrophage.

FRAbs were initially discovered in birth defects such as CFD, NTDs, and fetal malformation, which could partially explain why normal folate status aberrantly came alongside relative local folate deficiency in these cases. Recently, the roles of FRAbs in early pregnancy and miscarriage have been reported; however, since preliminary studies were only conducted using polyclonal antiserum against placental FRs (mainly consisting of FRα and FRβ) or FBP, the pathogenesis of miscarriage associated with FRAbs has not been deeply explored. In this review, we summarized the existing classical viewpoints of FRAbs and inferred several hypotheses regarding the generation of FRAbs during the periconceptional period. As emerging research on the expressional profile of FRs at the maternal-fetal interface accumulates, we attempt to discuss the potential roles of FRAbs, with regard to each specific FR isoform, in pregnancy maintenance and miscarriage. FRα mainly localizes in trophoblast cells, epithelia, and the stroma of decidua at the maternal-fetal interface; as such, FRαAb might interfere with trophoblastic biofunctions, placental development, decidualization, and decidual homeostasis. FRβ is a key signature of dMφ. Autoantibodies against FRβ might correlate with the origin of dMφ and angiogenesis, vascular remodeling, and immune regulation mediated by dMφ (Figure 3). FRγ and FRδ are secreted or expressed by neutrophils and Treg, respectively, but their functions at the maternal-fetal interface have rarely been studied, and their expressional profiles and phenotypes still need further verification.

FRAbs could potentially present a new strategy for predicting pregnancy outcomes. The efficacy of FRAbs in predicting subfertility has already been verified by preliminary studies [27]. Furthermore, combined with a newly developed noninvasive technology that retrieves and isolates EVTs from the cervix [204], it may be possible to evaluate the risks that FRAbs pose to the biofunctions of trophoblast cells in vitro. This could allow for tailored management or diagnostic algorithms for pregnant women with FRAbs. In addition, previous attempts to treat FRAb-positive birth defects may offer insights into dealing with reproductive failures complicated by positive FRAbs. Supplementary folate or its metabolically active derivatives can circumvent either an impaired intracellular folate-dependent enzyme pathway or an inhibitor of the cellular uptake of folate, likely via the compensatory effects mediated by other folate transporters [205]. Two forms of folinic acid (levofolinate and d,l-folinic acid) and 5-MTHF appear to accumulate better in the placenta and fetus than folic acid in the context of FRαAb [132,206]. Appropriate doses of corticosteroids (for instance, dexamethasone) might also rescue adverse pregnancy outcomes by alleviating autoimmune responses [110]; however, further exploration is required to determine the safe dosages of corticosteroids and their compatibility with other classical drugs used to treat pregnancy complications.

## Figures and Tables

**Table 1:** Expressional profile of FRs at the maternal-fetal interface.

Isoforms of FRs	GPI Anchor (Yes/No)	Species	Tissue	Localization of FRs Expression	References
FRα	Yes	Human	Placenta	syncytiotrophoblast (apical surface)	[29,84,104,105,106,107,108]
			Decidua	decidual glandular epithelial cells, decidual stromal cells	
		Mouse	Placenta	syncytiotrophoblasts, trophoblast giant cells, glycogen trophoblast cells and their progenitors	[109]
		Rat	Placenta	syncytiotrophoblasts, cytotrophoblasts	[26,110]
			Decidua	decidual cells	
FRβ	Yes	Human	Decidua	decidual monocytes/macrophages	[7,84,108]
FRγ	No	Human	(?)	secreted by neutrophils (?)	[111,112,113,114]
FRδ	Yes	Human/Mouse	Decidua (?)	decidual Treg cells (?)	[115,116,117]

(?) indicates that the expressional profile of FRs has not been confirmed at the maternal-fetal interface. Abbreviations: GPI anchor, glycosylphosphatidylinositol anchor.

**Table 2:** Evidence for implicating the association between FRAbs and miscarriage.

Ref.	Year	Subjects	Methods	Findings/Conclusions
[26]	2003	Rat	Intraperitoneal injection of antiserum to FRs at GD8	Application of FRs antiserum increased embryo absorption. Absorption rate induced by low dose of antiserum could be rescued by folinic acid. Absorption rate induced by large dose of antiserum cloud only be reserved by dexamethasone
[27]	2009	Human	Case-control study included in subfertility women (*n* = 17) and healthy controls (*n* = 25)	Women with blocking FRAbs showed a 12-times higher risk of subfertility
[135]	2015	Human	Case report of a FRAbs-positive woman with recurrent pregnancy losses	After the treatment of folate supplementation along with milk-free diet and prednisone to reduce the titer of FRAbs, she then conceived and had a successful pregnancy.
[133]	1983	Human	Retrospective study included in 280 sibships	There exist pairwise relationships among the NTD, FRAbs and miscarriage Maternal titer of FRAbs was elevated in NTD pregnancy. Higher titers of FRAbs increased risk of NTD pregnancy. Women with NTD pregnancy history showed higher risk of miscarriage. Recurrent miscarriage patients tended to conceive NTD-affected offspring.
[134]	2002	Human	Case-control study included in cases of positive maternal serum screening results for Down syndrome (*n* = 189) and matched controls (*n* = 945)	
[137]	2004	Human	Case-control study enrolled in women of NTD-pregnancy (*n* = 12) and controls (*n* = 24)	
[85]	2008	Human	Observational study collected maternal serum during 15–18th week of pregnancy	
[91,92]	2015	Mouse	Mouse fed with folate-free diet for 5 weeks before mating	Risk factor involving in FRAbs production were correlated with miscarriage Dysregulated methylation in decidua resulted in embryo losses

Abbreviations: GD, gestational day; NTD, neural tube defects.

**Table 3:** Characteristics or functions of *FOLR2*^+^ macrophages.

Characteristics/Functions	Source of Macrophages	Details	References
Differentiation	Differentiate from hematopoietic stem cells	FOLR2 was upregulated during differentiation and maturation Removal of FRAbs could alleviate the inhibitory effect on the precursor of macrophage	[171,172]
Recruitment	Recruit from peripheral monocytes	*FOLR2*^+^ macrophage may produce CCL2, CCL3 and CCL4 to facilitate its own enrichment in situ	[127,173,174,175]
Polarization	Induced from monocytes by M-CSF	M2-like macrophage was FOLR2-positive and depended on M-CSF	[176]
	Lung macrophages/Monocyte-derived macrophages	2. FOLR2 involved in maintaining M2-like phenotype and increasing folate uptake in the context of pro-inflammatory cytokines (like LPS or INF-γ)	[177,178]
		3. Repolarization from M2-like to M1-like phenotypes mediated by GM-CSF leaded to downregulated FOLR2, which could be rescued by anti-inflammatory treatment	[7,178,179,180]
		4. FRAbs targeting FOLR2 inhibited M2-mediated proliferation and fibrosis	[126,181]
Pro-angiogenesis	TAMs (Breast/lung cancer)	*FOLR2*^+^ TAMs resided near blood vessels and produced TGF-β and VEGF	[182,183]
	HBCs	2. HBCs was FOLR2-positive and derived from placental stroma 3. HBCs secreted VEGFA and may interact with decidual macrophages and trophoblasts via osteopontin	[127,184]
Immune regulation	TAMs (Hepatocellular/colorectal tumor, melanoma)	The crosstalk between *FOLR2*^+^ TAMs and Treg cells via CD86/CTLA4 or IL-10 could mediate immunotolerance	[176,185,186]
	Synovial macrophages	2. *FOLR2*^+^ macrophage might inhibit the activation of CD4^+^ T cells and NK cells by co-expressing CD39/CD73 and releasing immunosuppressive adenosine	[187,188]
	Peritoneal macrophages	3. Targeting IL-10 receptor impair the immune tolerance by downregulating FOLR2, tilting the functional balance towards M1-like phenotype and inhibiting vascular remodeling	[189]
	TAMs	4. Targeting *FOLR2*^+^ on TAMs re-polarized M2 to M1 and increased the infiltration of CD8+ T and pro-inflammatory monocytes	[190,191,192]
	HBCs	5. *FOLR2*^+^ macrophage highly expressed Toll-like receptor, implying its anti-microbial potency	[127]
	TAMs (Breast cancer)	6. *FOLR2*^+^ TAMs were MHCII-positive and presented a mixture of both M1 and M2 signatures 7. *FOLR2*^+^ TAMs colocalized and interacted with CD8+ cytotoxic T cells to activate antitumor response	[182]

## Data Availability

Not applicable.

## Abbreviations

CFD: cerebral folate deficiency; DHF: dihydrofolate; EVTs: extravillous trophoblasts; FR(s): folate receptor(s); FRAbs: autoantibodies against folate receptors; FOCM: folate-mediated one-carbon metabolism; GPI: glycosyl-phosphatidylinositol; Hcy: homocysteine; HTL: homocysteine thiolactone; NTD: neural tube defects; PCFT: proton-coupled folate transporter; RFC: reduced folate carrier; Met: methionine; THF: tetrahydrofolate; MS: methionine synthase; 5-MTHF: 5-methyltetrahydrofolate; 5,10-methylene THF: 5,10-methylenetetrahydrofolate.
